# Stage-dependent stoichiometric homeostasis and responses of nutrient resorption in *Amaranthus mangostanus* to nitrogen and phosphorus addition

**DOI:** 10.1038/srep37219

**Published:** 2016-11-16

**Authors:** Huiyuan Peng, Yahan Chen, Zhengbing Yan, Wenxuan Han

**Affiliations:** 1 Beijing Key Laboratory of Biodiversity and Organic Farming, Key Laboratory of Plant-Soil Interactions, Ministry of Education, College of Resources and Environmental Sciences, China Agricultural University, Beijing 100193, China; 2 Guizhou Institute of Biotechnology, Guiyang 550003, China; 3 Institute of Botany, Chinese Academy of Sciences, Beijing 100093, China; 4 Department of Ecology, College of Urban and Environmental Sciences, Peking University, Beijing 100871, China

## Abstract

Stoichiometric homeostasis is the ability of plants remaining their element composition relatively stable regardless of changes in nutrient availability, via various physiological mechanisms. Nutrient resorption is one of such key mechanisms, but whether and how nitrogen and phosphorus homeostasis and resorption in plants would change with growth-stages under variable nutrient supply was unclear. A nitrogen (N) and phosphorus (P) fertilizer addition experiment was conducted to evaluate the dynamics of N and P homeostasis and resorption efficiency during different growth-stages of *Amaranthus mangostanus* in a greenhouse. The homeostasis regulation coefficient of green-leaf P varied significantly, while that of green-leaf N maintained relatively stable across growth stages. Moreover, homeostasis regulation coefficient of N was higher at seedling stage but lower at flowering stage than that of P at corresponding stages, suggesting that the growth of *A. mangostanus* may switch from being more N- to P-limited from vegetative to reproductive stage. N resorption efficiency (NRE) was higher and P resorption efficiency (PRE) was lower at flowering than seed-filling stage. The nutrient dynamics displayed here suggested contrasting nutrient homeostasis and resorption responses of plants to environmental nutrient availability across growth stages. These findings can improve the understanding of nutrition maintenance mechanism of plants during their growth.

Nitrogen (N) and phosphorus (P) are the major limiting nutrients for plant growth in most ecosystems globally^1^,^2^,^3^. N and P availabilities greatly affect plant development. The most important influences of N on plant growth include modifying the number and size of organs and balancing the vegetative and reproductive growth. N shows stronger effects on vegetative than reproductive growth^4^. P deficiency can influence leaf formation and their final shapes, restrict flower initiation and seed formation^4^,^5^,^6^,^7^. Overall deficiency of N and P can affect many aspects of plant growth, especially photosynthesis, sugar metabolism, carbohydrate allocation among plant organs and reproductive activities^8^. Because of the different physiological roles of these nutrients, demand for N *vs* P varies differentially during plant growth, showing stage-dependent N and P stoichiometry of plants^9^,^10^,^11^.

The stability of plant nutrients (stoichiometric homeostasis) can be indicated by the homeostatic regulation coefficient (*H*; see below Equ. 1), which reflects the ability of plants to maintain their nutrient composition relatively stable regardless of changes in the environmental nutrients. When a nutrient limits their growth, plants can use multiple physiological mechanisms to improve the availability and use efficiency of the limiting nutrient, maintaining the stability of the body nutrient levels and the associated functioning^12^. These mechanisms of nutrient conservation include excreting hydrogen ions or enzymes into the soil, changing the allocation of photosynthetic products (*e.g.* increasing root:shoot ratio to increase nutrient uptake from the soils)^3^,^4^, increasing seed nutrient content that promotes seedling establishment in nutrient-deficient habitats^13^, and remobilizing nutrients from senescent to burgeoning organs (nutrient resorption)^13^,^14^,^15^,^16^,^17^,^18^.

At individual level, the *H* could reflect the variant ability of contain nutrients at specific growth stages. The higher the value of *H* is, the more stable nutrient content (a higher level of stoichiometric homeostasis) plants have^12^,^18^,^19^,^20^,^21^,^22^. While research has shown that mature trees had different N resorption efficiency from seedlings^23^, stoichiometric characters change with growth stages, leaf age and root condition^24^. It is not clear whether and how N and P homeostasis and resorption in plants would change with growth stages under the alternations in nutrient availabilities.

Given the different roles that N and P play during plant growth and development, we hypothesized that, the homeostasis and resorption of green-leaf N decreased from vegetative to reproductive stages, on the contrary the homeostasis and resorption of green-leaf P increased from vegetative to reproductive stages; At each stage the resorption efficiency for both nutrients might decrease in response to fertilizer additions. Herein, we chose *Amaranthus mangostanus*, an annual forb, in a fertilizer-addition experiment to evaluate the above hypothesis on the dynamic nutrient homeostasis and resorption efficiency during plant growth.

## Results

### Variation in green-leaf N and P homeostasis across growth stages

There was no significant difference among stages in the concentration and homeostasis (*H*) of green-leaf N (averagely N_gr_ = 24.4 mg g^−1^, 25.2 mg g^−1^, and 25.7 mg g^−1^) and *H*_*N*_ (4.76, 3.03, and 4.35), for seedling, flowering and seed-filling stage, respectively; ANOVA test, *p* > 0.05). However, P_gr_ and homeostasis of green-leaf P (*H*_*P*_) varied greatly across three growth stages, the highest values (P_gr_ = 9.17 mg g^−1^, *H*_*P*_ = 7.14) at flowering stage and the lowest (P_gr_ = 2.55 mg g^−1^, *H*_*P*_ = 2.17) at seedling stage (ANOVA test, *p* < 0.05). Green-leaf N:P ratio was significantly higher at vegetative growth (seedling) stage (12.4) than later reproductive growth stages, but did not show significant difference between reproductive stages (2.8 and 4.2 for flowering and seed-filling stage, respectively; *p* > 0.05); The homeostasis regulation coefficient (*H*_*N:P*_) was significantly higher at flowering than seedling stage (4.72 *vs.* 1.81 for flowering and seedling stage, respectively; *p* < 0.05). *H*_*N*_ was significantly larger than *H*_*P*_ (4.76 *vs.* 2.17; *p* < 0.05) at seedling stage, but smaller than *H*_*P*_ (3.03 *vs.* 7.14; *p* = 0.01) at flowering stage. There was no significant difference in *H* value between N and P (*p* = 0.13) at seed-filling stage.

### Concentration and resorption responses to N and P addition across growth stages

**Figure 1:**
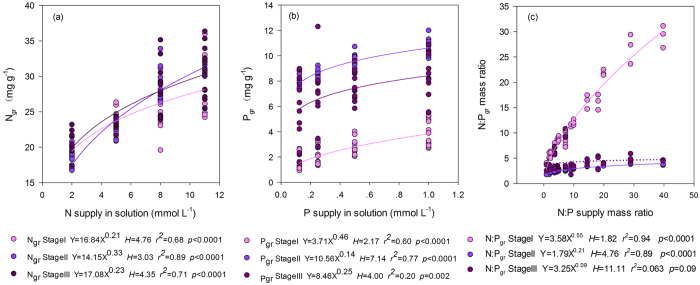
Mature green-leaf N and P concentrations under different levels of N and P supply across growth stages of *Amaranthus mangostanus*. The power regression was used to determine regression lines. (**a**) relationship between N concentration in mature green-leaf (N_gr_) and N supply; (**b**) relationship between P concentration in mature green-leaf (P_gr_) and P supply; (**c**) relationship between N:P mass ratio of mature green-leaf (N:P_gr_) and N:P ratio of supply in solution.

**Figure 2:**
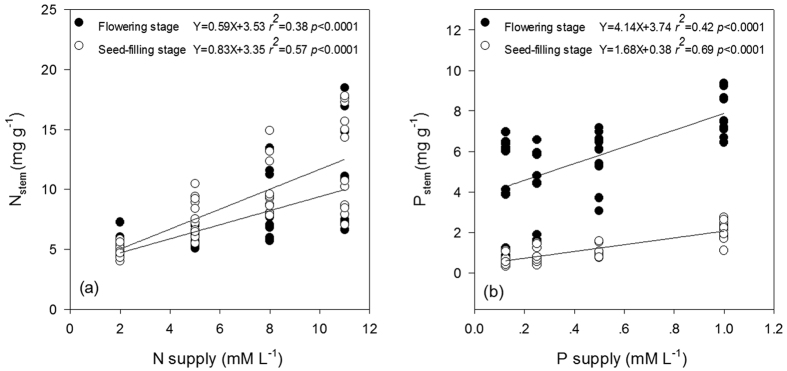
Relationship between N/P supply *vs* N_stem_ and P_stem_ of flowering stage and seed-filling stage in *Amaranthus mangostanus*. (**a**) N supply *vs* N_stem_; (**b**) P supply *vs* P_stem_. Linear regression was used to determine regression lines.

Addition of N or P significantly increased green-leaf N (N_gr_) or P (P_gr_) (Fig. 1a,b) and stem N (N_stem_) or P (P_stem_) (Fig. 2a,b) concentration at all stages. Green-leaf N:P (N:P_gr_) was significantly affected by solution N:P (N:P_supp_) (Fig. 1c).

**Figure 3:**
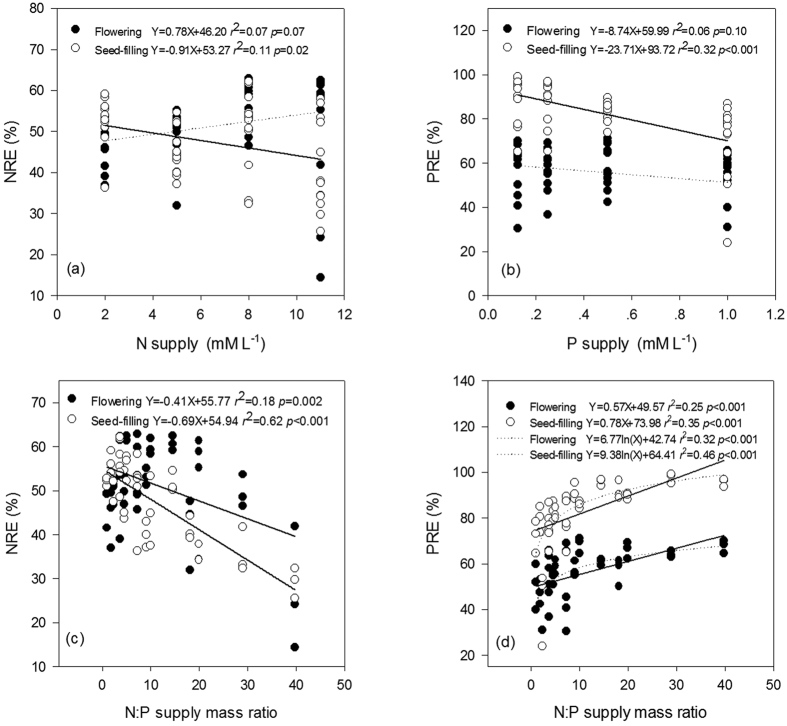
Relationship between N/P supply and N:P supply mass ratio *vs* NRE and PRE of different stages in *Amaranthus mangostanus*. (**a**) N supply *vs* NRE; (**b**) P supply *vs* PRE; (**c**) N:P supply mass ratio *vs* NRE; (**d**) N:P supply mass ratio *vs* PRE. Linear regression was used to determine regression lines.

Nutrient resorption in *A. mangostanus* occurred only during the reproductive growth stages when senesced-leaves appeared. Addition of N or P significantly decreased the nutrient resorption efficiency (NuRE) at seed-filling stage (Fig. 3a,b). P_stem_ was significant higher at flowering stage than at seed-filling stage (Fig. 2b), however N_stem_ did not show significant difference between the two stages.

With the increase of N:P_supp_, *i.e.*, N supply increased relatively more than P supply, N resorption efficiency (NRE) was reduced (Fig. 3c) and P resorption efficiency (PRE) was increased (Fig. 3d). PRE was significantly higher at seed-filling (82.6%) than flowering (55.9%) stage (ANOVA test, *p* < 0.05). On the contrary, there was no significant difference in NRE between flowering (51.3%) and seed-filling (47.3%) stage (ANOVA test, *p* > 0.05).

**Figure 4:**
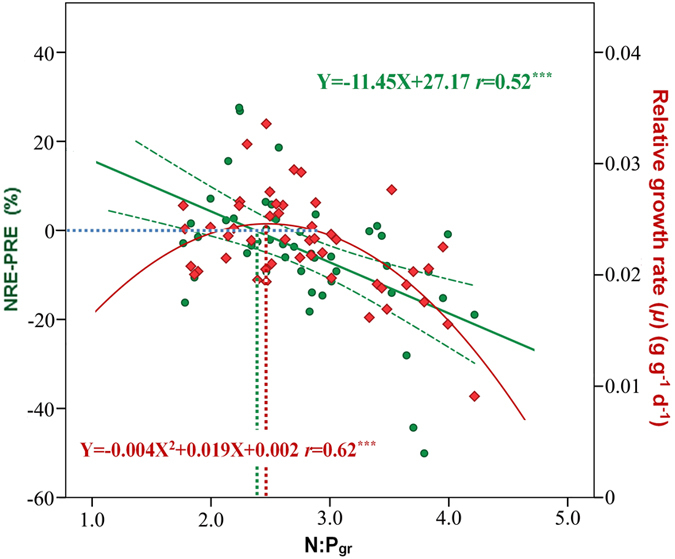
Relationship between N:P_gr_ *vs* relative nutrient resorption efficiency (NRE-PRE) and relationship between relative growth rate (*μ*) *vs* N:P_gr_ at flowering stage of *Amaranthus mangostanus*.

Moreover, the relative resorption efficiency of N *vs* P (NRE - PRE, denoted as RR hereafter) was negatively correlated with N:P_gr_ (Fig. 4). The corresponding N:P_gr_ was 2.37 (95%CI: 1.85–2.66) at flowering stage, when RR ≈ 0 (NRE ≈ PRE).

During the flowering period, the relative growth rate (*μ*) of *A. mangostanus* reached its peak value at N:P_gr_ = 2.38 (Fig. 4); this N:P_gr_ ratio was nearly equal to the predicted value of N:P_gr_ (2.37) according to the hypothesis of relative resorption efficiency.

## Discussion

### Plant N and P stoichiometric homeostasis reflects nutrient status at different stages

Nitrogen regulates the development of plant organs and exerts stronger control on vegetative growth than reproductive growth^4^. At the early stage of growth (vegetative growth stage before flowering), *A. mangostanus* thus need to maintain the stability of N_gr_ more than P_gr_ to guarantee a healthy vegetative growth, which was reflected by the stoichiometric character of higher *H*_*N*_ and lower coefficient of variance (CV_N_) compared with *H*_*P*_ and CV_P_ at the same stage (*H*_*N*_ = 4.76 > *H*_*P*_ = 2.17; CV_N_ = 16.8% < CV_P_ = 44.8%; Fig. 1a,b; Table 1).

**Figure 5:**
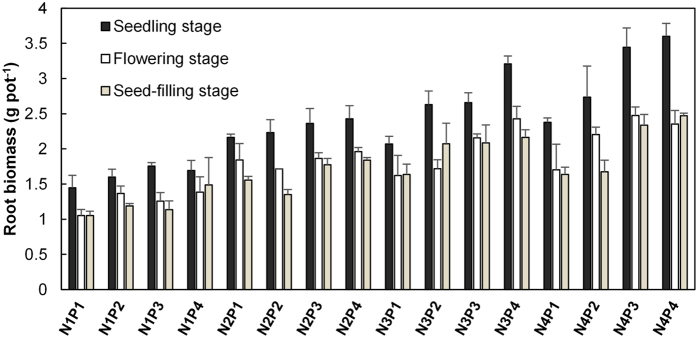
Root biomass of *Amaranthus mangostanus* under different fertilization regimes at three growth stages.

Phosphorus regulates florescence and seed formation^4^, and turns into the crucial nutrient at the reproductive growth stages. The root biomass significantly decreased at flowering stage and seed-filling stage, compared with seedling stage (Fig. 5). The P remobilized (resorbed) from senescent organs becomes the main source of P at this period^25^. In cereal plants, up to 90% of cereal grains total-P was remobilized from vegetative organs^4^. Therefore, *A. mangostanus* may need a steady and plentiful supply of P to support their reproductive growth after flowering, showing very high P_gr_, P_stem_ and *H*_*P*_ at this time. Furthermore, the flourishing growth and high photosynthetic rate at flowering stage also demand for high and stable leaf P_gr_ concentration^3^. Similar variation pattern of P_gr_ was observed in other plants (*e.g*. In Lettuce^26^).

The principle that was demonstrated by the variations in green-leaf N and P concentration and homeostasis was consistent with the dynamic physiological demand for the two nutrients at different growth stages. *H*_*N*_ > *H*_*P*_ at seedling stage and *H*_*P*_ > *H*_*N*_ at flowering stage, which reflected the relative importance of the roles played by N and P at different (vegetative *vs* reproductive) stages. Thus, the N_gr_ *vs* P_gr_ homeostasis dynamics across the growth stages of *A. mangostanus* accorded with the stability of limiting elements hypothesis that the limiting nutrient in plants usually shows low variability and environmental sensitivity^27^.

### Plants resorbed P more than N at reproductive stages

The plants had a large demand for the nutrients, especially for P, examined in the reproductive growth stage because of the biomass increment and the corresponding nutrient accumulation, and the emergence of reproduction organs such as flowers and seeds^4^. Because of the decrease of root biomass nutrient uptake by roots probably couldn’t maintain a stable nutrient supply for plant growth at this stage, so nutrient resorption mechanism was a critical strategy for the homeostasis of plant N and P during reproductive stages. At least 50% of the leaf N and P were recycled through nutrient resorption^17^,^28^,^29^. In this study, 51.3% leaf N and 55.9% leaf P were reused at flowering stage, and up to 82.6% of leaf P was reused at seed-filling stage. 21.3% stem N and 34.0% stem P were reused at seed-filling stage. N_stem_ did not show significant difference between flowering stage and seed-filling stage. The variation in stem nutrient concentration was in accordance with the variance in NuRE (Fig. 2). We couldn’t determine the mass loss of leaves because the leaves of this species were thin and became seriously shrunk during senescence. Thus, the NuRE calculated here may have somewhat underestimation^29^. However, the underestimation shouldn’t affect the RR values and the comparison results between stages, because the relative resorption efficiency (RR) is the difference between NRE and PRE which has nothing to do with leaf mass loss (see Equ. 2 & 4 17,18), and because the between-stage comparisons were under the same condition without leaf mass-loss compensation.

PRE > NRE at both of the reproductive stages. The resorption mechanism was expected to contribute the homeostasis of P in the reproductive organs (flowers and seeds). The fact that P accumulation in reproductive organs of *A. mangostanus* was significantly higher at seed-filling than flowering stage (30.2 *vs.* 26.8 mg pot^−1^; *p* < 0.05; Table S1) further suggested that more P needed to be resorbed from leaves and stems to meet the higher and stable demand for P^3^,^13^ at seed-filling stage. Thus PRE was significantly higher at seed-filling than flowering stage (Fig. 3d) and the P_stem_ was significantly lower at seed-filling stage than flowering stage (Fig. 2b).

In this study, leaves and stems of *A. mangostanus* show similar dynamics in nutrient re-translocation. Both leaf P and stem P were re-used at seed-filling stage when large amount of P were demanded. But it is still unclear how different in nutrient re-translocation between organs is in annual versus perennial plants.

### Relative more than absolute nutrient limitation controlled nutrient resorption at different stages

Nutrient resorption from senesced plant tissues is a key nutrient conservation strategy^30^,^31^. Plants grown in poor soils are expected to show high nutrient resorption. Yuan and Chen^31^ revealed that NuRE of N and P declined in response to respective nutrient fertilization in general via a meta-analysis on a global dataset of fertilization experiments. However, another independent meta-analysis based on more than 60 fertilization experiments found that only about 1/3 of the cases showed negative relationships between NuRE and fertilization, whereas NuRE was not responsive, or was even responded positively, to fertilizer addition in the other cases^32^. In this experiment, NuRE (NRE and PRE) were negatively related to fertilization (N_supp_ and P_supp_ respectively) only at seed-filling stage, but had no significant relationship with nutrient supply at flowering stage (see Fig. 3a,b). However, given that the growth of plants are limited by not only the absolute supply of N and P but also their relative availability (indicated here by N:P_supp_), the relative resorption hypothesis^17^ suggested that NuRE should respond to N:P_supp_ in a consistent way across both growth stages. And this was just what our results (Fig. 3c,d) demonstrated: NRE decreased and PRE increased with increasing N:P_supp_ (N in surplus and P in shortage, relatively) at both flowering and seed-filling stages.

### Optimal green-leaf N:P at vegetative and reproductive growth stage

Optimal/critical N:P_gr_ varies across growth stages and among different species^33^. N:P_gr_ is an important indicator of plant nutrient status: plants tended to be P-limited at low N:P_gr_, or N-limited at high N:P_gr_^33^. For example, Yan *et al*.^34^ reported that the critical N:P_gr_ of *Arabidopsis thaliana* was around 16 at flowering stage. Fertilizer experiments suggested that the optimal N:P_gr_ was 4–6 for above 40% cereal and oil crops which obtained maximum yield but the optimal ratio for grain legumes achieving maximum yield was about 9 at their mature stage^35^.

In this study, the N:P_gr_ ratio (19.2) corresponding to the maximal relative growth rate (*μ*_max_) (Fig. S2) was significantly greater than the mean N:P_gr_ (12.4; *p* < 0.05) at the early vegetative growth (seedling) stage, suggesting a general N-limitation on the growth of *A. mangostanus* under the current fertilizing regime at this stage.

However, the N:P_gr_ at *μ*_max_ (the so-called optimal or critical N:P_gr_ ratio) was 2.38 for *A. mangostanus* at flowering stage (Fig. 4), close to the lower limit for grains and oilseed crops^35^. Moreover, relative growth rate increased with N:P_gr_ when N:P_gr_ was lower than 2.38 (N-limited), but decreased with N:P_gr_ when N:P_gr_ was higher than 2.38 (P-limited). Further analysis using relative resorption hypothesis^17^ (RRH) confirmed this optimal (critical) N:P_gr_ for *A. mangostanus* at flowering stage. RRH supposed that plants tended to resorb proportionately more of the limiting nutrient (here it is P), resulting in a negative relationship between the difference in the proportionate resorption of N *vs* P (i.e., NRE minus PRE, or RR) and foliar N:P_gr_^17^. Actually RR of *A. mangostanus* showed significantly negative relationship with N:P_gr_ (Fig. 4). Moreover, the RR values, mostly below zero, indicated that the growth of *A. mangostanus* was mainly restricted by P during the reproductive growth stages (Fig. 4). A previous study on *Amaranth* species suggested that *A. mangostanus* was a kind of phosphorophilous (P-like) plant^36^. As shown in this experiment, the N:P_gr_ threshold (around 2.4) of *A. mangostanus* at the mature (flowering) stage was markedly lower than the previous reported optimal N:P value (15) for most terrestrial plants^1^,^33^, given that the high P demand by P-like species.

### Conclusion

Leaf N homeostasis (and the concentration) did not change significantly across growth stages of *A. mangostanus*, but leaf P homeostasis (and the concentration) was higher at reproductive stages than vegetative stage. N showed more stable homeostasis than P at vegetative (seedling) stage, but the opposite was true at the flowering stage, given the different physiological function of N and P in plant growth.

Nutrient resorption efficiency for both N and P decreased with the relative availability of these two nutrients (indicated by medium N:P_supp_ ratio, or the green-leaf N:P_gr_) at flowering stage, consistent with the relative resorption hypothesis. NRE was higher and PRE was lower at flowering than seed-filling stage. The N and P dynamics displayed here suggested contrasting nutrient homeostasis and resorption responses of plants to environmental nutrient availability across growth stages. The optimal green-leaf N:P ratio may change with plant growth stages, suggesting that plant growth may switch from being more N- to P-limited from vegetative to reproductive stage. These findings can improve the understanding of nutrition maintenance mechanism of plants during their growth, and help optimize fertilization management, but further research is required to test the applicability to more species with various life histories and growth-forms.

## Materials and Methods

### Material

*Amaranthus mangostanus* (colored amaranth), an annual forb of Amaranthaceae, is widely distributed in China. This plant is rich in vitamins, calcium, phosphorus, iron and other nutrients, which makes it a common vegetable. Seeds of this commercially vegetable cultivar was obtained from Chinese Academy of Agricultural Sciences. *A. mangostanus* can be clearly distinguished between vegetative and reproductive growth stages (see Fig. S1), with very small seeds of 0.5–1.0 mg averagely^37^.

### Experiment condition and design

A pot experiment was conducted in a greenhouse at China Agricultural University in 2014. Seeds were surface sterilized by soaking them in a 1% potassium permanganate solution for 10 min and then rinsing with distilled water. After accelerating germination in dark for 48 h, seeds were sown in pots which were filled with 50% vermiculite and 50% perlite in early May. The diameter of the pot was 22 cm and the depth was 16 cm. Two weeks after germination, the redundant seedlings were removed until five individuals were left in each pot. We adopted a two-way full-factorial design which included four N levels (2, 5, 8, 11 mM N L^−1^, added as NH_4_NO_3_) and four P levels (0.125, 0.25, 0.5, 1 mM P L^−1^, added as KH_2_PO_4_ and NaH_2_PO_4_), totally 16 treatments (Table 2). These solutions were based on Hoagland’s formula and previous studies^34^ and were modified according to the experimental subject and aims of this study. All pots were supplied with the same concentrations of macro- and micro-elements (0.75 mM L^−1^ K_2_SO_4_, 0.65 mM L^−1^ MgSO_4_, 1 μM L^−1^ MnSO_4_, 0.1 μM L^−1^ CuSO_4_, 1 μM L^−1^ ZnSO_4_, 0.035 μM L^−1^ Na_2_MoO_4_, 0.1 mM L^−1^ Fe-EDTA, 0.01 mM L^−1^ H_3_BO_3_, and 2 mM L^−1^ CaCl_2_) except for N and P. The pH of each solution was adjusted to 6.5. Each pot was watered with 300 ml nutrient solution of the respective treatment every three or four days. To minimize the effects of micro-environmental conditions, the pots were randomly rearranged every 15 days.

### Sampling and assay methods

We harvested our plants at three stages. The first stage was seedling stage before first flower buds appeared (65 days after germination); the second stage, defined as flowering stage, lasted until all flowers disappeared (104 days after germination); and the third stage, seed-filling stage, was the period when seeds matured (122 days after germination). Three replicate pots were harvested for each treatment over the three stages. Green leaves, senesced leaves, roots and other organs (such as stems, flowers and seeds) were sampled from each pot. Above-ground biomass were obtained by pooling the biomass of foliar, stem and reproductive organs together.

The collected samples were oven dried for 48 h at 70 °C. Dried samples were measured to calculate biomass and then powdered using a mortar and pestle. Plant nutrient concentrations were measured after H_2_SO_4_–H_2_O_2_ digestion. Tissue N concentration was determined colorimetrically by the Kjedahl acid-digestion method with an Alpkem autoanalyzer (Kjektec System 1026 Distilling Unit, Sweden). Tissue P concentration was measured by the phosphorus vanadium-molybdate yellow colorimetric method.

### Data analysis

The plant nutrient stoichiometry response to environment nutrient levels over the three growth stages were estimated by calculating the homeostatic regulation coefficient (*H*) according to the following equation^21^:


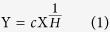


where Y was the N or P concentration (mg g^−1^) or N:P mass ratio of mature green leaves, X was the N or P concentration or N:P mass ratio of the nutrient solutions and *c* was a constant.

Nutrient resorption efficiency (NuRE) was calculated as follows:


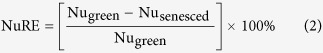


where Nu_green_ and Nu_senesced_ were the nutrient (N/P) concentrations per dry weight (mg g^−1^) in mature green leaves and senesced leaves, respectively.

Aboveground biomass (AB) included the biomass of mature green leaves, senesced leaves and other organs.

The relative growth rate (*μ*)^21^ was calculated using biomass only at the seedling and flowering stages, excluding the seed-filling stage due to the small difference in biomass between the seed-filling stage and flowering stage. *μ* was calculated as following equation:


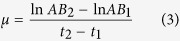


where AB_1_ and AB_2_ are the aboveground biomass at seedling stage and flowering stage (g) respectively, t_1_ is the number of days from sowing to the first of the harvest (65d), and t_2_ is the number of days from sowing to the second of the harvest (104d). 1000-seeds weight (0.7 g) was adopted^37^ to calculate the biomass (0.0035 g) at the beginning of germination.

Relative nutrient resorption efficiency (RR) was calculated as following equation:


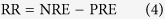


where NRE and PRE are the N resorption efficiency and P resorption efficiency, respectively. Relative nutrient resorption hypothesis suggests that plants resorb proportionately more N or P (RR > 0 or <0) when they are N (or P) limited, or similar proportions of N and P (RR ≈ 0) when co-limited by both nutrients^17^.

Differences among the effects of N, P and the N × P interactions were determined using two-way analysis of variance (ANOVA). Power regression was performed to assess the relationship between mature green-leaf nutrients and nutrient addition levels (i.e. N_gr_ against N_supp_; P_gr_ against P_supp_; N:P_gr_ against N:P_supp_). Linear regression was performed to assess the relationship between nutrient resorption efficiency and nutrient addition levels (i.e. NRE against N_supp_ and N:P_supp_; PRE against P_supp_ and N:P_supp_), and between N:P_gr_ and RR with *μ*. Statistical analyses were performed using the software IBM SPSS Statistics 20 (2011, ver. 20; SPSS Inc., USA).

## Additional Information

**How to cite this article**: Peng, H. *et al*. Stage-dependent stoichiometric homeostasis and responses of nutrient resorption in *Amaranthus mangostanus* to nitrogen and phosphorus addition. *Sci. Rep.* **6**, 37219; doi: 10.1038/srep37219 (2016).

**Publisher’s note:** Springer Nature remains neutral with regard to jurisdictional claims in published maps and institutional affiliations.

## Supplementary Material

Supplementary Information

## Figures and Tables

**Table 1:** Statistics of N_gr_/P_gr_, NRE/PRE, NRE-PRE (RR), homeostasis regulation coefficient (*H*) and their coefficients of variation (CV, %) at different growth stages of *Amaranthus mangostanus*.

Growth stage	N_gr_		P_gr_		NRE		PRE		RR		N:P_gr_		*H*_*N*_	*H*_*P*_
	Mean (mg g^−1^)	CV	Mean (mg g^−1^)	CV	Mean (%)	CV	Mean (%)	CV	Mean (%)	CV	Mean	CV		
Seedling	24.4	16.8	2.6	44.8	—	—	—	—	—	—	12.4	61.6	4.8	2.2
Flowering	25.2	21.8	9.2	12.9	51.3		55.9		−4.6	−305.1	2.8	23.1	3.0	7.1
Seed-filling	25.8	18.4	7.1	32.8	47.3	19.8	82.6	17.1	−37.6	−49.7	4.2	44.5	4.4	4.0
Whole growth	25.1		6.3		49.3		69.2		−21.1		6.4		4.1	4.4

**Table 2:** N and P concentrations and N:P mass ratio in solutions of the 16 treatments (four N levels: N1, N2, N3, N4; and four P levels: P1, P2, P3, P4).

N supply level	P supply level	N (mM L^−1^)	P (mM L^−1^)	N:P mass ratio
N1	P1	2	0.125	7.23
	P2	2	0.25	3.61
	P3	2	0.5	1.81
	P4	2	1	0.90
N2	P1	5	0.125	18.06
	P2	5	0.25	9.03
	P3	5	0.5	4.52
	P4	5	1	2.26
N3	P1	8	0.125	28.90
	P2	8	0.25	14.45
	P3	8	0.5	7.23
	P4	8	1	3.61
N4	P1	11	0.125	39.74
	P2	11	0.25	19.87
	P3	11	0.5	9.94
	P4	11	1	4.97
