# Supplementary material for: Stage-dependent stoichiometric homeostasis and responses of nutrient resorption in Amaranthus mangostanus to nitrogen and phosphorus addition
Source: Sci Rep. 2016 Nov 16;6:37219. doi: 10.1038/srep37219 (PMC5110967; doi:10.1038/srep37219)

Stage-dependent stoichiometric homeostasis and responses of nutrient resorption in *Amaranthus mangostanus* to nitrogen and phosphorus addition

Huiyuan Peng[1,2]， Yahan Chen[3]， Zhengbing Yan[4]， Wenxuan Han [1*]

[1] Beijing Key Laboratory of Biodiversity and Organic Farming, Key Laboratory of Plant-Soil Interactions, Ministry of Education, College of Resources and Environmental Sciences, China Agricultural University, Beijing 100193, China

[2] Guizhou Institute of Biotechnology，Guiyang 550003，China

[3] Institute of Botany, Chinese Academy of Sciences, Beijing 100093, China

[4] Department of Ecology, College of Urban and Environmental Sciences, Peking University, Beijing 100871, China

Table S1 N and P accumulation in flowers and seeds during reproductive stages (paired sample *t*-test) of *Amaranthus mangostanus*.

Treatments	N (mg pot^{-1}) Flowering stage	N(mg pot^{-1}) Seed-filling stage	P(mg pot^{-1}) Flowering stage	P(mg pot^{-1}) Seed-filling stage
N1P1	56.8	76.9	8.2	11.3
N1P2	64.1^{a}	88.2^{b}	11.1	13.8
N1P3	59.8^{a}	78.8^{b}	13.9	18.3
N1P4	62.5	101.4	16.4	21.8
N2P1	127.0^{a}	136.8^{b}	12.6	11.2
N2P2	130.9	122.3	17.9	16.1
N2P3	154.6	190.9	33.3	35.5
N2P4	142.3^{a}	193.5^{b}	36.7	40.0
N3P1	115.6	172.5	11.5	11.9
N3P2	190.4^{a}	244.5^{b}	27.8^{a}	29.2^{b}
N3P3	213.1^{a}	277.7^{b}	38.9	41.1
N3P4	224.5	277.6	53.5	54.5
N4P1	157.4	181.5	18.1	14.5
N4P2	238.0	238.4	22.2	24.6
N4P3	301.3^{a}	374.9^{b}	43.7^{a}	55.3^{b}
N4P4	306.8^{a}	464.1^{b}	63.9^{a}	83.4^{b}
Mean	159.1	201.2	26.8^{A}	30.2^{B}

Values followed by the different capital letters refer to significant (p<0.05) difference within the flowering stage and seed-filling stage of all treatments

Values followed by the different lowercase letters refer to significant (p<0.05) difference within same treatment of two stages.

Table S2 Effects (F value) of N and P supply and their interaction(N×P) on total biomass, green-leaf N and P concentrations (N_{gr}/P_{gr}), senesced-leaf N and P concentrations (N_{sen}/P_{sen}), N and P resorption efficiency (NRE/PRE), relative resorption (NRE-PRE) (RR) of three different stages. The *F*-values and *p* values (***p<0.001; **p<0.01, *p<0.05) were determined by two-way analysis of variance

	Factors （df）	Total biomass (g)	N_{gr} （mg．kg^{-1}）	P_{gr} （mg．kg^{-1}）	N_{sen} （mg．kg^{-1}）	P_{sen} （mg．kg^{-1}）	NRE (%)	PRE (%)	RR (%)
Stage Ⅰ	N(3)	172.342***	86.980***	138.937***	-	-	-	-	-
	P(3)	21.004***	8.726***	227.410***	-	-	-	-	-
	N*P(9)	6.870***	6.939***	3.204**	-	-	-	-	-
Stage Ⅱ	N(3)	481.671***	527.441***	27.709***	31.309***	31.248***	9.264***	52.204***	15.224***
	P(3)	119.649***	13.751***	154.722***	31.931***	48.976***	17.599***	9.597***	23.923***
	N*P(9)	26.005***	4.563**	1.846	9.486***	8.863***	5.656***	8.630***	11.785***
Stage Ⅲ	N(3)	783.815***	130.761***	21.251***	150.072***	6.576**	14.834***	20.905***	14.090***
	P(3)	254.621***	15.073***	8.827***	98.193***	84.467***	29.360***	29.477***	129.233***
	N*P(9)	68.493***	9.279***	2.171	23.221***	0.998	3.976**	8.985***	6.955***

Table S3 Green-leaf biomass and nutrient(N/P) concentration of different stages

Treatments	Seedling stage			Flowering stage			Seed-filling stage		
	Green-leaf biomass	Green-leaf N concentration	Green-leaf P concentration	Green-leaf biomass	Green-leaf N concentration	Green-leaf P concentration	Green-leaf biomass	Green-leaf N concentration	Green-leaf P concentration
N1P1	1.36	19.80	2.56	1.70	18.60	7.41	1.79	19.73	2.04
N1P2	1.53	18.17	3.09	1.65	19.11	8.46	1.96	20.02	3.00
N1P3	1.49	19.31	3.61	1.53	17.07	9.05	1.45	22.43	6.95
N1P4	1.47	20.52	5.14	1.68	18.60	10.22	1.90	20.29	6.30
N2P1	2.59	23.63	1.47	3.02	22.08	7.69	2.94	23.75	5.93
N2P2	2.47	24.54	2.15	2.98	22.07	8.10	2.74	22.96	8.61
N2P3	2.44	24.39	2.85	3.23	21.68	8.90	3.27	23.43	7.53
N2P4	2.70	24.78	4.29	3.04	22.23	10.06	3.45	24.00	8.38
N3P1	3.07	27.16	1.02	3.14	32.16	8.04	3.31	32.95	6.97
N3P2	3.73	24.57	1.52	3.80	29.43	8.69	4.90	28.49	6.97
N3P3	3.53	23.88	2.37	4.82	28.37	10.16	5.72	24.22	7.20
N3P4	4.21	23.88	3.20	4.66	26.77	11.12	5.45	26.51	8.30
N4P1	3.50	33.70	1.16	3.55	32.49	8.75	3.25	35.21	7.77
N4P2	4.04	30.71	1.40	4.01	31.95	8.75	2.88	31.87	7.97
N4P3	5.46	26.35	2.18	5.88	29.51	9.94	7.06	28.18	8.90
N4P4	5.14	24.76	2.80	6.08	31.02	11.47	8.16	28.02	10.47
Mean	3.05	24.38	2.55	3.42	25.20	9.18	3.76	25.75	7.08

Table S4 Senesced-leaf biomass and nutrient(N/P) concentration of different stages

Treatments	Flowering stage			Seed-filling stage		
	Senesced-leaf biomass	Senesced -leaf N concentration	Senesced -leaf P concentration	Senesced -leaf biomass	Senesced -leaf N concentration	Senesced -leaf P concentration
N1P1	0.17	9.64	4.52	0.18	10.36	0.53
N1P2	0.21	9.68	4.63	0.31	9.16	0.79
N1P3	0.31	9.50	4.71	0.52	9.76	1.36
N1P4	0.37	9.92	5.04	0.68	9.71	1.71
N2P1	0.38	12.88	3.29	0.61	13.95	0.41
N2P2	0.49	10.32	3.44	0.80	13.77	0.66
N2P3	0.42	10.81	4.08	0.83	12.23	1.25
N2P4	0.34	11.16	7.41	0.41	11.84	4.17
N3P1	0.93	16.23	2.89	1.68	21.09	0.29
N3P2	0.93	11.55	3.40	1.95	13.71	0.28
N3P3	0.74	11.10	3.26	1.02	10.94	0.84
N3P4	1.06	10.75	4.17	1.05	10.80	1.73
N4P1	1.08	23.77	2.83	2.27	24.92	0.35
N4P2	0.74	13.25	2.95	2.46	20.54	0.81
N4P3	1.40	11.82	3.13	1.94	15.27	1.25
N4P4	1.38	11.85	4.74	2.00	12.39	1.88
Mean	0.68	12.14	4.03	1.17	13.78	1.14

Table S5 Flowers & seeds biomass and N/P concentration of different stages

Treatments	Flowering stage			Seed-filling stage		
	Flowers & seeds biomass	Flowers & seeds N concentration	Flowers & seeds P concentration	Flowers & seeds biomass	Flowers & seeds N concentration	Flowers & seeds P concentration
N1P1	2.22	25.60	3.67	2.73	28.26	4.11
N1P2	2.56	25.10	4.32	3.16	27.95	4.36
N1P3	2.76	21.69	5.03	2.99	26.37	6.13
N1P4	2.60	24.17	6.33	3.64	27.91	5.97
N2P1	4.44	28.64	2.84	4.51	30.30	2.48
N2P2	4.94	26.56	3.63	4.10	29.94	3.93
N2P3	5.58	27.82	5.98	6.55	29.15	5.41
N2P4	5.23	27.23	7.02	6.95	27.84	5.76
N3P1	3.54	32.56	3.26	5.08	33.94	2.35
N3P2	5.86	32.49	4.74	7.22	33.93	4.06
N3P3	6.97	30.62	5.61	8.94	31.17	4.63
N3P4	7.74	28.99	6.89	9.37	29.63	5.81
N4P1	4.48	35.19	4.01	5.31	34.19	2.74
N4P2	6.95	34.24	3.19	6.67	35.88	3.72
N4P3	9.74	30.93	4.48	12.13	30.93	4.58
N4P4	9.94	30.93	6.44	15.53	29.89	5.36
Mean	5.35	28.92	4.84	6.55	30.45	4.46

Fig. S1 *Amaranthus mangostanus* at (a) seedling stage and (b) flowering stage in this experiment.

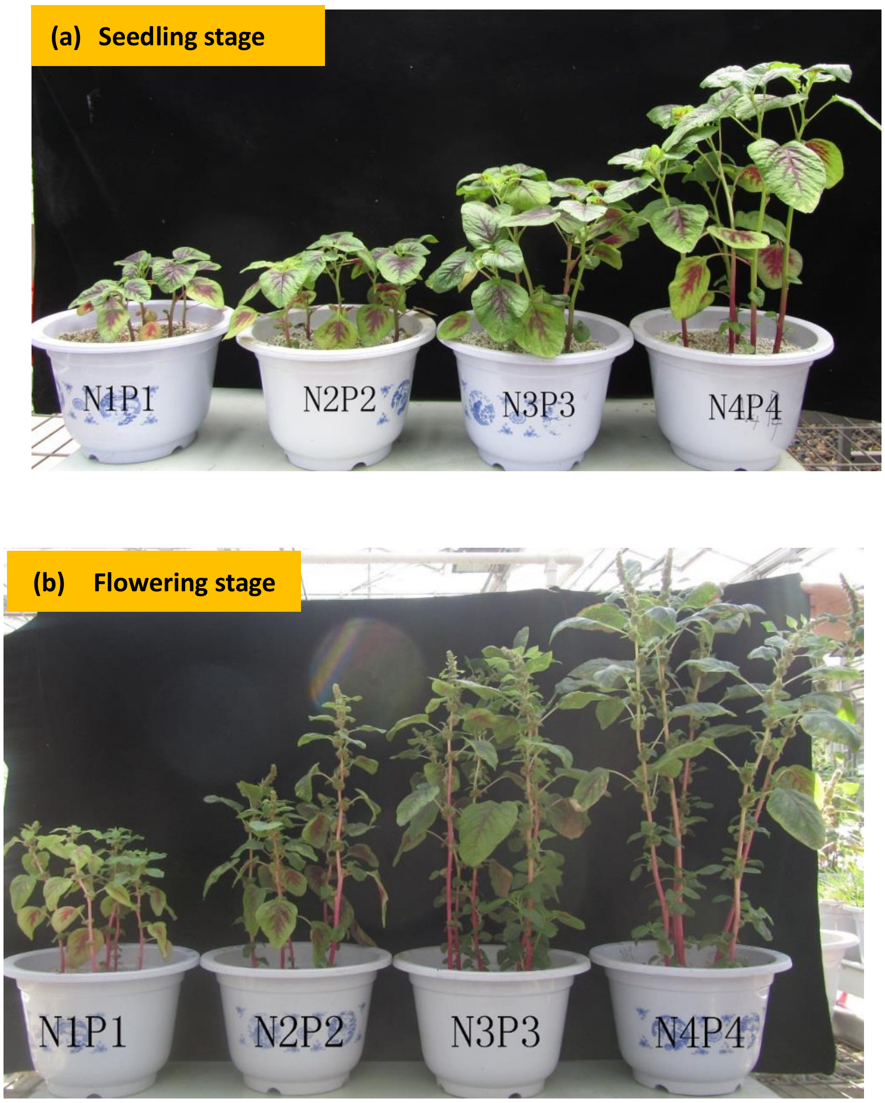

Fig. S2 Relationship between relative growth rate (μ) and N:P$_{gr}$ at seedling stage of *Amaranthus mangostanus*.

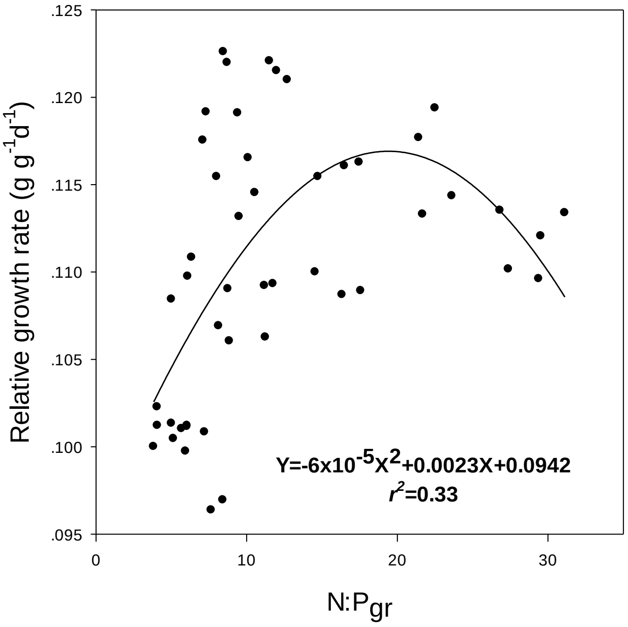

Supplement: Supplementary Information [file srep37219-s1.pdf]
